# Targeting Epstein–Barr virus oncoprotein LMP1-mediated glycolysis sensitizes nasopharyngeal carcinoma to radiation therapy

**DOI:** 10.1038/onc.2014.32

**Published:** 2014-03-24

**Authors:** L Xiao, Z-y Hu, X Dong, Z Tan, W Li, M Tang, L Chen, L Yang, Y Tao, Y Jiang, J Li, B Yi, B Li, S Fan, S You, X Deng, F Hu, L Feng, A M Bode, Z Dong, L-q Sun, Y Cao

**Affiliations:** 1 Cancer Research Institute, Xiangya School of Medicine, Central South University, Changsha, China; 2 Key Laboratory of Carcinogenesis and Cancer Invasion, Ministry of Education, Changsha, China; 3 Key Laboratory of Carcinogenesis, Ministry of Health, Changsha, China; 4 The First Hospital of Changsha City, Changsha, China; 5 Metabolic Syndrome Research Center, The Second Xiangya Hospital, Central South University, Changsha, China; 6 Center For Molecular Medicine, Xiangya Hospital, Central South University, Changsha, China; 7 Clinical Biochemical Laboratory, Xiangya Hospital, Central South University, Changsha, China; 8 Pathology Department, Xiangya Hospital, Central South University, Changsha, China; 9 Pathology Department, The Second Xiangya Hospital, Central South University, Changsha, China; 10 Department of Radiation Oncology, Emory University School of Medicine, Winship Cancer Institute of Emory University, Atlanta, GA, USA; 11 Endocrinology Department, The Second Xiangya Hospital, Central South University, Changsha, China; 12 The Hormel Institute, University of Minnesota, Austin, MN, USA

## Abstract

Our goal in this work was to illustrate the Epstein-Barr virus (EBV)-modulated global biochemical profile and provide a novel metabolism-related target to improve the therapeutic regimen of nasopharyngeal carcinoma (NPC). We used a metabolomics approach to investigate EBV-modulated metabolic changes, and found that the exogenous overexpression of the EBV-encoded latent membrane protein 1 (LMP1) significantly increased glycolysis. The deregulation of several glycolytic genes, including hexokinase 2 (HK2), was determined to be responsible for the reprogramming of LMP1-mediated glucose metabolism in NPC cells. The upregulation of HK2 elevated aerobic glycolysis and facilitated proliferation by blocking apoptosis. More importantly, HK2 was positively correlated with LMP1 in NPC biopsies, and high HK2 levels were significantly associated with poor overall survival of NPC patients following radiation therapy. Knockdown of HK2 effectively enhanced the sensitivity of LMP1-overexpressing NPC cells to irradiation. Finally, c-Myc was demonstrated to be required for LMP1-induced upregulation of HK2. The LMP1-mediated attenuation of the PI3-K/Akt-GSK3beta-FBW7 signaling axis resulted in the stabilization of c-Myc. These findings indicate a close relationship between EBV and glycolysis in NPC. Notably, LMP1 is the key regulator of the reprogramming of EBV-mediated glycolysis in NPC cells. Given the importance of EBV-mediated deregulation of glycolysis, anti-glycolytic therapy might represent a worthwhile avenue of exploration in the treatment of EBV-related cancers.

## Introduction

In order to sustain unlimited growth, most tumor cells consume glucose through the glycolytic pathway as their major energy source to rapidly generate ATP even in the presence of oxygen.^1^ This phenomenon, called the Warburg effect, has been consistently observed in most cancers, and is considered to be an emerging hallmark of cancer.^2^ Oncogenic viruses, which are estimated to cause 11.9% of human cancers, have been reported to modify several cellular metabolic pathways.^3^ Thus, by integrating metabolomics analysis results with the preexisting atlas of gene expression in cancer cells, some significant interactions between metabolite variations and infection-related carcinogenesis could be discovered and present new opportunities for the regimen of oncogenic virus-related cancer treatment.

Epstein-Barr virus (EBV), the first characterized oncogenic virus,^4^ is highly prevalent in humans and is estimated to cause about 1% of all human cancers, including B-cell lymphoma, nasopharyngeal carcinoma (NPC) and gastric carcinoma.^5^ EBV encodes a series of functional proteins and non-coding RNAs, which orchestrate two types of infection, latent and lytic. Latent membrane protein 1 (LMP1) is essential for the maintenance of latent infection and EBV-mediated malignant transformation. The large cytosolic C terminus of LMP1 provides docking sites for several signaling adapter proteins, which trigger various downstream oncogenic signaling pathways, such as the NF-κB, PI3-K/Akt and JAK/STAT pathways.^6^ In this way, LMP1 promotes malignant phenotypes, including resistance to apoptosis, and increased angiogenesis, invasion and metastasis in EBV-harboring cancer cells.^7, 8, 9^ However, the relationship between LMP1 and metabolic changes is still unclear.

To provide enough energy for carcinogenesis, many adaptive mechanisms have been shown to be involved in the metabolic reprogramming of cancer cells. The canonical oncogenic signaling pathways, including PI3-K/Akt and c-Myc, have been reported to reprogram the core energy metabolism by regulating vital metabolic enzymes or transporters, such as hexokinase 2 (HK2), lactate dehydrogenase A and cellular transporters, leading to greater nutrient uptake and increased macromolecular biosynthesis.^10,11^ In this study, using a metabolomics approach, we illustrate a profile of EBV-mediated metabolic changes. Very importantly, HK2 was identified as a key modulator of LMP1-induced glycolysis, and conferred proliferative advantages and poor prognosis of NPC patients following radiation therapy. c-Myc was confirmed to be crucial for LMP1-induced upregulation of HK2 and glycolysis, and the LMP1-perturbed PI3-K/Akt-GSK3beta-FBW7 axis resulted in the stabilization of c-Myc. These findings suggest that targeting metabolic enzymes is particularly critical for EBV-related tumor cell proliferation and survival and might be a novel therapeutic pursuit.

## Results

### Epstein-Barr virus-encoded LMP1 changes the metabolic profile and promotes increased glycolysis in NPC cells

**Figure 1:**
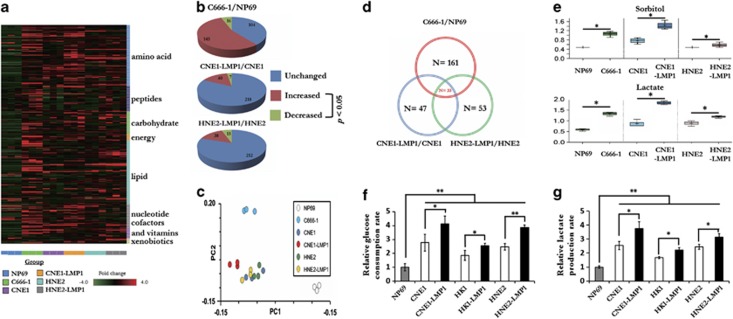
Epstein-Barr virus-encoded LMP1 changes the metabolic profile and promotes elevation of the glycolytic pathway in NPC cells. (**a**) Heat map showing 265 biochemical factors in lysates from three replicates each of immortalized nasopharyngeal epithelial cells (NP69 cells), EBV-positive NPC cells (C666-1 cells), EBV-negative NPC cells (CNE1 and HNE2) and LMP1-overexpressing cells (CNE1-LMP1 and HNE2-LMP1). The relative fold change for each biochemical factor in each sample is represented as a relative mean value increase (red) or decrease (green). (**b**) Pie charts indicating the number of biochemical factors with mean levels that are significantly (*P*<0.05, Welch's *t*-test) higher or lower in EBV-positive NPC cells compared with immortalized nasopharyngeal epithelial cells, or in LMP1-overexpressing NPC cells compared with LMP1-negative NPC cells. (**c**) Venn diagrams indicating the number of biochemical factors with mean levels that are significantly (*P*<0.05) higher or lower in C666-1, CNE1-LMP1 and HNE2-LMP1 cells compared with the NP69, CNE1 and HNE2 cells. (**d**) Principal components analysis of the metabolite profile data set. The percentage of variance in the data set reflected by the first six principle components is shown in the histogram. PC1 and PC2 for each sample are plotted. (**e**) The cellular levels of the sorbitol pathway components, sorbitol and lactate, were detected in three pairs of cell lines. (**f**, **g**) The effect of LMP1 on glycolytic flux in NPC cells. The levels of glucose consumption (**f**) and lactate production (**g**) were examined in NP69 cells, NPC cell lines (CNE1, HK1, HNE2 cells) and LMP1-overexpressing NPC cells (CNE1-LMP1, HK1-LMP1, HNE2-LMP1 cells) using the Automatic Biochemical Analyzer (#7170A, HITACHI, Japan). Data are shown as means±s.d. of three experiments. The asterisks indicate a significant (*P<0.05 and **P<0.001) difference.

To assess the metabolic changes triggered by EBV infection, we used a metabolomics approach to analyze differences among the global metabolic profiles of six different cell lines. These cell lines included immortalized nasopharyngeal epithelial cells (NP69 cells), EBV-positive NPC cells (C666-1 cells), EBV-negative NPC cells (CNE1 and HNE2) and LMP1-overexpressing NPC cells (CNE1-LMP1 and HNE2-LMP1). The analysis revealed mass spectrum ion counts corresponding to 265 known biochemical compounds. We normalized these data to protein concentration and produced a heat map of each biochemical group in its appropriate classification (Figure 1a). The general changes in the global metabolic profiles among the various cell lines are shown as pie charts (Figure 1b). Principal components analysis results showed that cells with EBV infection or LMP1 expression could be distinguished from controls (NP69, CNE1 and HNE2 cells) by their principal component 1 value (27.81% of variance), and the presence of both EBV infection and LMP1 expression resulted in an obvious different biochemical profile (Figure 1c).

Thirty-three biochemical factors were altered in the same direction in C666-1 cells, CNE1-LMP1 and HNE2-LMP1 cells compared with NP69 and their respective parental cells (Figure 1d). Interestingly, the levels of sorbitol and lactate were significantly elevated. Compared with NP69 cells, the level of sorbitol in C666-1 cells was much higher (Figure 1e), and LMP1-overexpressing NPC cells also exhibited higher levels of sorbitol compared with their respective parental counterparts. More importantly, lactate levels were substantially higher in C666-1 cells and LMP1-overexpressing NPC cells compared with NP69 and their respective parental counterparts. When the glycolytic pathway is activated and glucose uptake is elevated, sorbitol is generated mostly through the reduction of glucose, and the final product of glycolysis, lactate, increases. Therefore, we hypothesized that glycolysis might be upregulated by EBV and LMP1.

To further confirm our metabolomics data, we examined the level of glucose consumption and lactate production in three pairs of NPC cell lines. Consistent with the results of the metabolic profile analysis, LMP1-overexpressing CNE1-LMP1, HK1-LMP1 and HNE2-LMP1 cells consumed more glucose (Figure 1f) from the media and extruded a greater amount of lactate (Figure 1g) into the media compared with the respective parental CNE1, HNE2 and HK1 cells. Knocking down LMP1 expression significantly suppressed the glucose consumption and lactate production rate in C666-1 cell (Supplementary Figures 1A–2B). These findings combined with the global metabolic profile analysis reflect a greater glycolytic flux in LMP1-overexpressing NPC cells and suggest a vital role of LMP1 in EBV-mediated glucose metabolic reprogramming of NPC cells.

### HK2 is a key modulator of LMP1-mediated elevated glycolysis

To investigate the mechanisms of the LMP1-mediated elevation of glycolysis and disruption of glucose metabolic reprogramming, qRT–PCR screening was performed to evaluate the expression profiles of key glucose metabolism-related genes in NP69 cells and multiple NPC cell lines. As shown in Supplementary Figure 2, results indicated that *hk2* and other several glycolytic genes were significantly more highly transcribed in C666-1 and LMP1-overexpressing NPC cells compared with NP69 and LMP1-nonexpressing NPC cells, respectively.

**Figure 2:**
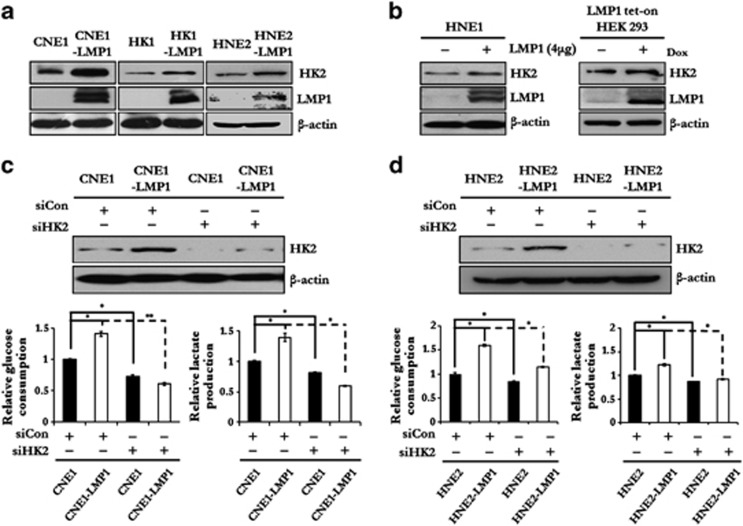
HK2 is a key modulator of LMP1-mediated glycolysis. (**a**, **b**) The effect of LMP1 on the protein expression level of HK2 was determined by immunoblot analysis in LMP1 transiently transfected HNE1 and LMP1 tet-on HEK293 cells. β-Actin was used as a control to confirm equal loading of protein. (**c**, **d**) HK2 expression in CNE1, CNE1-LMP1, HNE2 and HNE2-LMP1 cells was knocked down by siHK2 and the relative levels of glucose consumption and lactate production rate were examined in these cell lines using the Automatic Biochemical Analyzer. Data are shown as means±s.d. of three experiments. The asterisks indicate a significant (*P<0.05 and **P<0.001) difference.

HK2, a rate-limiting enzyme of glycolysis, is upregulated in multiple cancers. Immunoblot analysis indicated that the HK2 protein level increased in response to LMP1 expression (Figures 2a and b and Supplementary Figure 1A). To further assess the role of HK2 in LMP1-mediated glycolysis, we knocked down HK2 expression and then examined the relative glucose consumption rate and lactate production rate in NPC cells. While the knockdown of HK2 impaired the glycolytic rate in all tested NPC cells, the inhibitory efficacy was significantly higher in LMP1-expressing cells (Figures 2c, d). Similar results were also observed in LMP1-knockdown C666-1 cells (Supplementary Figure 1D). Based on these data, we proposed that LMP1-modulated upregulation of several glycolytic genes might be the molecular basis responsible for the LMP1-mediated high glycolysis level.

### Upregulation of HK2 by LMP1 confers NPC cells with a proliferative advantage and resistance to apoptosis

**Figure 3:**
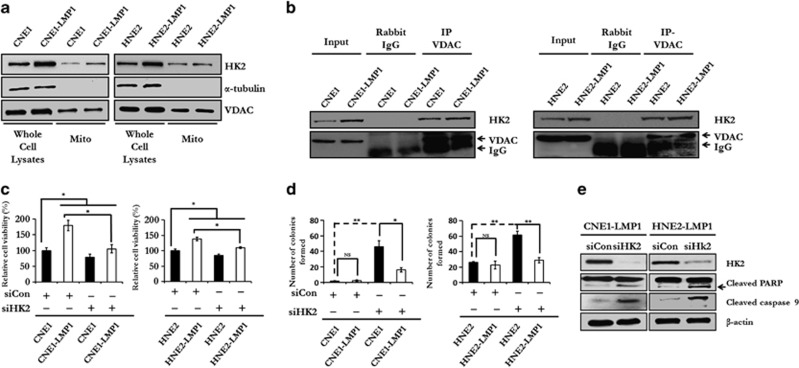
LMP1 promotes the translocation of HK2 to the mitochondrial outer membrane and increases HK2-mediated anti-apoptotic activity in the NPC cells. (**a**) The effect of LMP1 on the translocation of HK2 to the mitochondrial outer membrane was determined by immunoblot analysis in each of two pairs of NPC cell lines, including CNE1 cells compared with CNE1-LMP1 cells and HNE2 cells compared with HNE2-LMP1 cells. α-Tubulin served as an internal control to verify equal loading of whole-cell lysates. VDAC served as an internal control to confirm equal loading of mitochondrial fractions. (**b**) An immunoprecipitation assay was performed to test the interaction of HK2 and VDAC in LMP1-expressing cells and their parental cells. (**c**) An MTS assay was performed to evaluate the effect of HK2 on cell viability. Knocking down HK2 by siHK2 significantly impaired the proliferative rate of LMP1-overexpressing NPC cells relative to control cells. Data are shown as means±s.d. of three experiments. (**d**) The colony formation assay was executed to determine the effect of depleted HK2 on the colony formation ability of LMP1-overexpressing NPC cells. Data are shown as means±s.d. of three experiments. (**e**) HK2 expression in CNE1-LMP1 and HNE2-LMP1 cells was knocked down by siHK2 and the expression of full-length or cleaved caspase-9 and PARP was analyzed by immunoblotting. β-Actin served as internal control to confirm equal loading of proteins. The asterisks indicate a significant (*P<0.05 and **P<0.001) difference and NS indicates no significant difference.

In cancer cells, for HK2 to exert its anti-apoptotic function, HK2 translocates to the mitochondrial outer membrane and interacts with the voltage-dependent anion channel (VDAC) to block the release of cytochrome c, eventually inhibiting caspase-9-dependent apoptosis.^12,13^ To evaluate the influence of LMP1 on the intracellular localization of HK2, mitochondrial fractions from NPC cells, with or without LMP1 expression, were extracted and immunoblotted with antibodies to detect HK2, tubulin and VDAC. Results indicated that the translocation of HK2 in the mitochondrial outer membrane fraction was enhanced in both CNE1-LMP1 and HNE2-LMP1 cells (Figure 3a). Furthermore, immunoprecipitation assay results demonstrated that the interaction between VDAC and HK2 was much stronger in LMP1-expressing NPC cells (Figure 3b). We therefore hypothesized that if HK2 endowed tumor cells with a proliferative advantage and apoptotic resistance in response to EBV-encoded LMP1, knocking down the expression of HK2 might decrease glycolysis and survival advantage in LMP1-overexpressing cells. To test this hypothesis, we attenuated HK2 expression using small interfering RNAs (siRNAs) in CNE1, CNE1-LMP1, HNE2 and HNE2-LMP1 cells, and then examined cell proliferation by MTS and colony formation assays. Knocking down HK2 expression significantly impaired cell viability (Figure 3c) and colony formation efficiency (Figure 3d) of LMP1-overexpressing NPC cells, compared with control groups. We also examined caspase-9 and poly ADP ribose polymerase (PARP) expression after HK2 knockdown. The levels of cleaved of caspase-9 and PARP in CNE1-LMP1 and HNE2-LMP1 cells were markedly enhanced by downregulation of HK2 using siRNA (Figure 3e), indicating a crucial role of HK2 in anti-apoptotic regulation in LMP1-overexpressing cells. In this case, LMP1-mediated upregulation of HK2 promotes anti-apoptosis effects and increased proliferation of NPC cells.

### Upregulation of HK2 corresponds with poor overall survival of NPC patients

**Figure 4:**
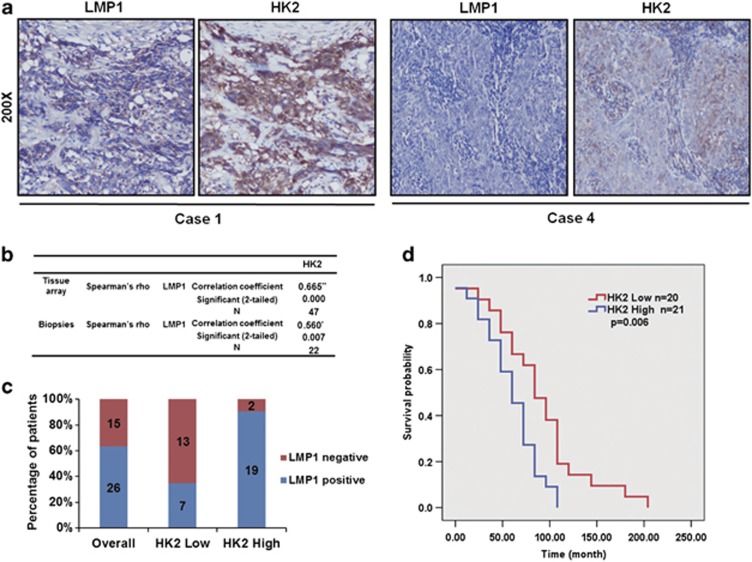
Upregulation of HK2 corresponds with poor overall survival of NPC patients. (**a**) Immunohistochemistry analysis was used to examine the level of HK2 and LMP1 expression in an NPC tissue array and tumor biopsies from NPC patients. In a biopsy from Case 1, both HK2 and LMP1 were highly expressed, whereas in Case 4, HK2 and LMP1 were expressed at low levels. (**b**) Correlation between HK2 and LMP1 expression in the NPC tissue array (correlation coefficient=0.665, *P*<0.0001) and NPC biopsies (correlation coefficient=0.560, *P*=0.007). (**c**) The positive frequencies of LMP1 in HK2 low expression and HK2 high-expression group are shown. (**d**) Overall survival rates of NPC patients with high (*n*=21) or low (*n*=20) expression levels of HK2 were estimated with the Kaplan–Meier method by log-rank test (*P*=0.006).

Given the crucial role of HK2 in regulating glycolysis and inhibiting apoptosis, we investigated whether LMP1-mediated upregulation of HK2 was associated with the prognosis of NPC patients. We examined the expression levels of HK2 and LMP1 in a commercial NPC tissue array and in additional biopsies from 22 NPC patients acquired from the Xiangya Hospital. The immunohistochemistry  analysis revealed a significant correlation between HK2 and LMP1 expression in both the NPC biopsies (correlation coefficient=0.560, *P*=0.007) and NPC tissue array (correlation coefficient=0.665, *P*<0.001) (Figures 4a and b, Supplementary Figure 3). Of the 60 patients whose tumor biopsies were used in the NPC tissue array, 47 patients were successfully followed up (at a median follow-up of 5.81 years) and their clinical characteristics are listed in Supplementary Table 3. Based on the clinical follow-up data of the NPC tissue array, we retrospectively analyzed the prognostic significance of HK2 expression in the overall survival (OS) of 41 NPC patients, who received radiation therapy or concomitant chemoradiotherapy. We divided these patients into two groups based on the expression level of HK2 and analyzed the correlation between HK2 expression level and the OS of these two patient groups. As shown in Figures 4c and 4d, results of the Kaplan–Meier method analysis with log-rank test revealed a statistically significant difference in the OS between the HK2 high-expression group of 21 patients (19 of 21 with expression of LMP1, median survival time=62.29 months) and the HK2 low-expression group of the other 20 patients (7 of 20 with expression of LMP1, median survival time=93.60 months). These results not only indicated that HK2 was upregulated along with LMP1, but also suggested that HK2 overexpression was associated with a worse clinical outcome of radiation therapy.

### Knockdown of HK2 sensitizes NPC cells to radiation therapy

**Figure 5:**
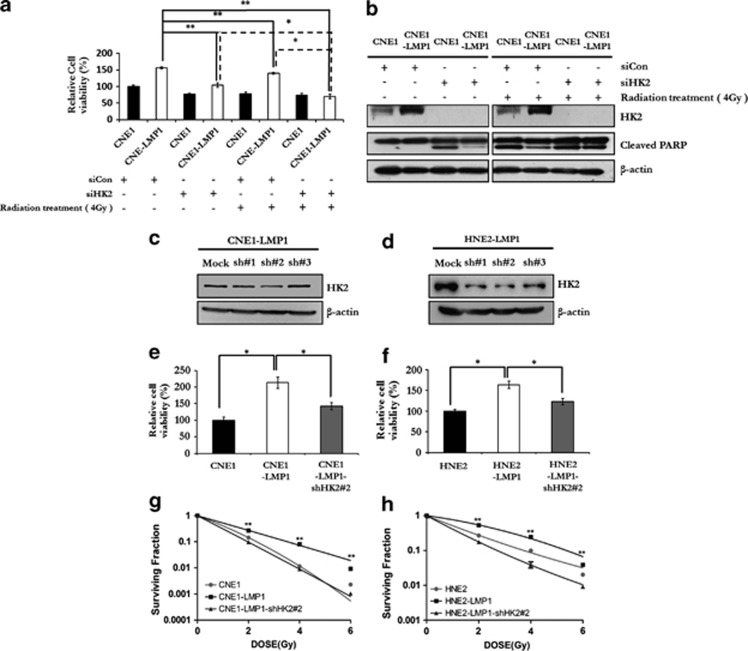
Knockdown of HK2 sensitizes NPC cells to radiation therapy. (**a**) Effect of HK2 depletion on the sensitivity of NPC cells to radiation therapy was measured by MTS assay. CNE1 and CNE1-LMP1 cells were transfected with siCon (control) or siHK2 and then treated with radiation. The relative inhibition was calculated by comparing the OD value of each treatment group with the control group (that is, CNE1-LMP1 cells transfected with siCon). Data are shown as means±s.d. of three experiments. (**b**) Immunoblotting analysis was performed to detect the level of HK2 and PARP in CNE1 and CNE1-LMP1 cells expressing siCon or siHK2 and exposed or not exposed to radiation treatment. β-Actin served as internal control to confirm equal protein loading. (**c**, **d**) Immunoblot analysis was performed to examine the HK2 protein level in the HK2-knockdown CNE1-LMP1 and HNE2-LMP1 cells and β-actin served as a control to verify equal protein loading. (**e**, **f)** The MTS assay was performed to examine the viability of CNE1, CNE1-LMP1, CNE1-LMP1-shHK2#2 cells, HNE2, HNE2-LMP1 and HNE2-LMP1-shHK2#2. Data are shown as means±s.d. of three experiments. (**g**, **h**) Colony formation of CNE1, CNE1-LMP1, HNE2 and HNE2-LMP1 cells with or without HK2 expression was evaluated 10 days after irradiation with a single dose of 0, 2, 4 or 6 Gy. Surviving fractions were calculated by comparing the colony number of each treatment group with untreated groups (0 Gy). Data are shown as means±s.d. of three experiments. The asterisks indicate a significant (*P<0.05 and **P<0.001) difference.

Because the clinical outcome of NPC patients with a high HK2 expression level is worse than that of those with a low level of HK2 expression, depleting HK2 from NPC cells might improve the outcome of radiation-based treatment. To test the effect of HK2 depletion on the sensitivity of NPC cells to radiation therapy, *siCon* or *siHK2* RNA was transfected into CNE1 and CNE1-LMP1 cells, and the cells were then irradiated. The results of an MTS assay indicated that radiation treatment could decrease the viability of all tested cell lines (Figure 5a). Notably, the viability of CNE1-LMP1 cells transfected with HK2 siRNA was significantly lower compared with the cells transfected with *siCon*. Strikingly, radiation treatment combined with HK2 siRNA decreased cell viability of CNE1-LMP1 cells with a synergistic effect (*P*<0.05). In contrast, no such effect was detected in CNE1 cells, implying a synergistic effect of combining HK2 depletion and irradiation in killing LMP1-overexpressing NPC cells. To further confirm our hypothesis, immunoblot analysis was performed to detect HK2 and PARP in NPC cells with or without irradiation after HK2 siRNA transfection. As shown in Figure 5b, the expression of the HK2 protein was almost completely abolished after HK2 siRNA transfection. The level of cleaved PARP dramatically increased in CNE1 cells with or without radiation treatment after HK2 siRNA transfection. On the other hand, the level cleaved PARP level was just slightly enhanced in CNE1-LMP1 cells by HK2 siRNA transfection alone or radiation treatment alone, but substantially increased after the combined treatment. In addition, we found that LMP1-overexpressing NPC cells and C666-1 were more sensitive to the glycolysis inhibitor, 2-deoxyglucose, compared with their counterpart cells. Similarly, radiation treatment combined with 2-DG treatment synergistically decreased cell viability of LMP1 overexpressing and C666-1 cells (*P*<0.05, Supplementary Table 4 and Supplementary Figure 4).

A radiosensitivity assay was performed to evaluate the radiosensitivity of NPC cells after stable knockdown of HK2. By using lentiviral-based HK2 small hairpin RNA (shRNA), stable HK2 knockdown cell pools of CNE1-LMP1 and HNE2-LMP1 cells were constructed. Immunoblot analysis demonstrated that HK2 levels were attenuated in these cell pools. Viability was also decreased in HK2-depleted cells (Figures 5c and f). The radiosensitivity of NPC cells with or without HK2 expression was evaluated after irradiation at a single dose of 0, 2, 4 or 6 Gy. In CNE1-LMP1 cells, depletion of HK2 had a greater inhibitory effect on colony formation and resulted in a lower survival rate after irradiation compared with CNE1 cells (Figure 5g). Similar results were also obtained after depletion of HK2 in HNE2-LMP1 cells (Figure 5h). These data suggested that knockdown of HK2 could sensitize LMP1-overexpressing NPC cells to irradiation.

### c-Myc is required for LMP1-mediated upregulation of HK2 in NPC

**Figure 6:**
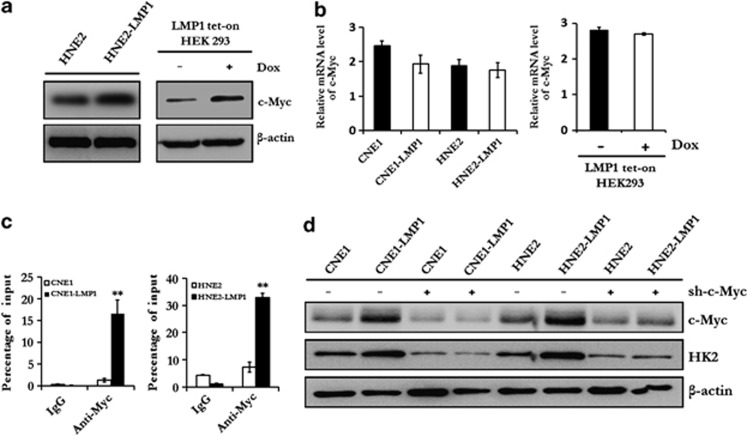
c-Myc is required for LMP1-mediated upregulation of HK2 in NPC. (**a**) Immunoblotting was used to analyze the expression level of c-Myc in HNE2, HNE2-LMP1 and LMP1 tet-on HEK293 cells. β-Actin served as an internal control to verify equal loading of proteins. (**b**) qRT–PCR was performed to determine the mRNA level of *c-Myc* in LMP1-overexpressing cells and their parental cells, and LMP1 tet-on HEK293 cells. β-Actin was used as a control to confirm equal loading of cDNAs. Data are shown as means±s.d. of three experiments. (**c**) A ChIP assay was performed with NPC cells (CNE1, CNE1-LMP1, HNE2 and HNE2-LMP1) using a c-Myc antibody and normal rabbit immunoglobulin G (IgG) to examine the binding of c-Myc to the *hk2* promoter region. Data are shown as means±s.d. of three experiments. (**d**) The effect of knocking down c-Myc expression (lentiviral c-Myc shRNA) on HK2 expression was determined by immunoblot analysis. β-Actin served as an internal control to verify equal loading of proteins. The asterisks indicate a significant (*P<0.05 and **P<0.001) difference.

c-Myc is known as a master regulator of energy metabolism.^1^ To determine whether c-Myc is responsible for LMP1-mediated upregulation of HK2, immunoblot analysis and qRT–PCR were performed to examine the expression level of c-Myc in LMP1-overexpressing cells or EBV-positive C666-1 and P3HR1 cells with knockdown LMP1 cells. Here, we found that LMP1 did not significantly affect the messenger RNA level of *c-myc* (Figure 6a and Supplementary Figure 1C), but strikingly affected the protein level of c-Myc (Figure 6b and Supplementary Figure 1A), which is consistent with previous results.^14^ Furthermore, a chromatin immunoprecipitation assay was performed to confirm that c-Myc was driven to the HK2 locus by LMP1 (Figure 6c), and knockdown of c-Myc significantly decreased the expression level of HK2 (Figure 6d). These data provided direct evidence that c-Myc was a key transcriptional factor of LMP1-mediated upregulation of HK2, whereas the expression of c-Myc was regulated by LMP1 at the post-transcriptional level.

### LMP1-mediated attenuation of the PI3-K/Akt-GSK3beta-FBW7 signaling axis results in the stabilization of c-Myc and upregulation of glycolysis

**Figure 7:**
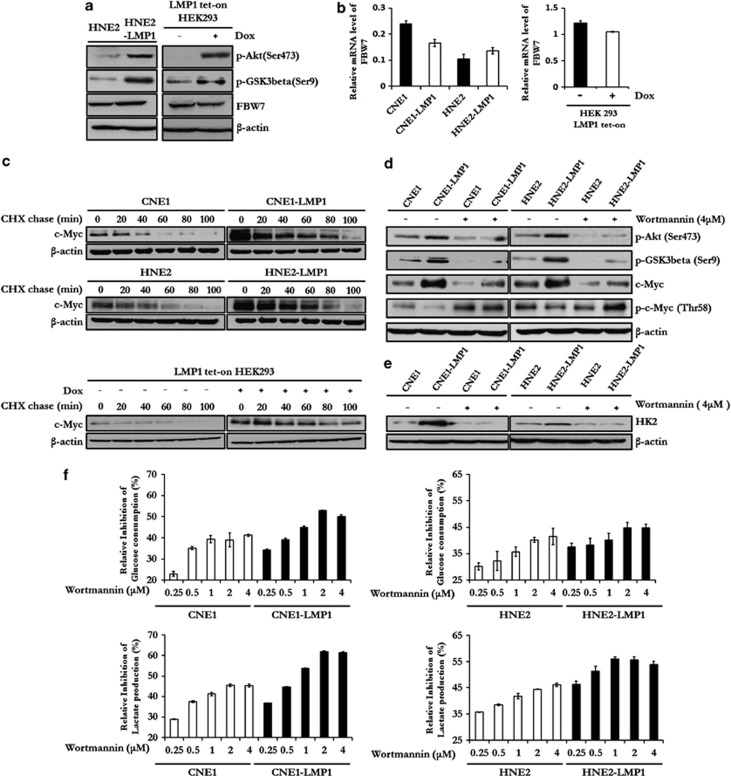
LMP1-mediated attenuation of the PI3-K/Akt-GSK3beta-FBW7 signaling axis results in stabilization of c-Myc and upregulation of glycolysis. (**a**) Immunoblotting was used to analyze the expression level of FBW7 and the phosphorylation level of Akt and GSK3beta in HEN2, HNE2-LMP1 and LMP1 tet-on HEK293 cells. β-Actin served as an internal control to verify equal loading of proteins. (**b**) qRT–PCR was performed to determine the mRNA level of *fbw7* in LMP1-overexpressing cells and their parental cells, and LMP1 tet-on HEK293 cells. β-Actin was used as a control to confirm equal loading of cDNAs. Data are shown as means±s.d. of three experiments. (**c**) The effect of LMP1 on stability of c-Myc in NPC cells and LMP1 tet-on HEK293 cells was examined by the cycloheximide chase assay, cells were treated with cycloheximide (20 μg/ml) for the indicated times. c-Myc expression levels were determined by immunoblot analysis. β-Actin served as an internal control to verify equal loading of proteins. (**d**) The effect of Wortmannin treatment on c-Myc expression and phosphorylation of Akt (Ser473), GSK3beta (Ser9) and c-Myc (Thr58) was determined by immunoblot analysis. β-Actin served as an internal control to verify equal loading of proteins. (**e**) The effect of Wortmannin treatment on HK2 expression was determined by immunoblot analysis. β-Actin served as an internal control to verify equal loading of proteins. (**f**) CNE1, CNE1-LMP1, HNE2 and HNE2-LMP1 cells were treated with Wortmannin at the indicated doses. The relative levels of glucose consumption and lactate production rate were examined in these cell lines using the Automatic Biochemical Analyzer. Data are shown as means±s.d. of three experiments.

Previous studies have demonstrated that the GSK3beta-FBW7 axis is crucial for the ubquitination-dependent degradation of several pivotal oncogenes, including *c-myc*, and GSK3beta is a key component of the PI3-K/Akt signaling pathway.^15^ In our investigation of the regulatory effect of the PI3-K/Akt signaling pathway and the GSK3beta-FBW7 axis on glycolysis in NPC cells, we found that the phosphorylation level of Akt (Ser473) and GSK3beta (Ser9) were increased by LMP1 in both LMP1-overexpressing NPC cells and LMP1 tet-on HEK293 cells, whereas the expression level of FBW7 was not affected (Figures 7a, b). Similar results were also observed in C666-1 and P3HR1 cells compared with their counterparts with knocked-down LMP1 expression (Supplementary Figure 1A). Furthermore, we found that LMP1 stabilized the c-Myc protein by extending its half-life (Figure 7c). To further probe the connection between PI3-K/Akt and c-Myc stabilization in the presence of LMP1, we used Wortmannin, a specific inhibitor of the PI3-K/Akt pathway, to treat NPC cells and examined whether or not c-Myc stabilization would be affected by the inhibition of the PI3-K/Akt signaling pathway in these cells. Results indicated that treatment with Wortmannin for 100 minutes dramatically decreased the phosphorylation level of Akt (Ser473), GSK3beta (Ser9) and the protein level of c-Myc in CNE1-LMP1 and HNE2-LMP1 cells, while increasing the phosphorylation level of c-Myc at Thr58, a site phosphorylated by GSK3beta and then targeted by FBW7 for degradation (Figure 7d). Although Thr58 and Ser62 of c-Myc are reportedly mutated in several cancers, our sequencing data showed that the *c-myc* gene was not mutated at either of these two sites in all tested cell lines (Supplementary Figure 5). Moreover, Wortamannin treatment also resulted in a reduction of HK2 expression in NPC cells and this inhibitory effect was more salient in CNE-LMP1 and HNE2-LMP1 cells (Figure 7e), leading to a much higher dose-dependent inhibition of the glycolytic flux compared with their respective parental cells (Figure 7f). Taken together, these data indicated that the PI3-K/Akt-GSK3beta-FBW7 signaling axis was required for LMP1-mediated upregulation of c-Myc, and thus promoted HK2 expression and elevated glycolysis in LMP1-expressing NPC cells.

## Discussion

EBV is widespread in the human population and etiologically linked to several types of cancers.^4^ Unlike other herpes viruses, EBV has a unique set of growth-activating genes and establishes a latent growth-transforming infection in host cells. LMP1 is essential for EBV-mediated growth transformation because of its activation of numerous oncogenic signaling pathways.^16^ Even though several studies reported that EBV and LMP1 might affect intercellular energy metabolism, an EBV-modulated global biochemical profile has not yet been published and the underlying mechanism has rarely been investigated.^17,18^ In our study, we used a large-scale metabolic profiling approach and found that EBV and LMP1 modulated metabolic changes in NPC cells and, in particular, elevated glycolysis. Importantly, the LMP1-mediated deregulation of several glycolytic genes revealed the potency of LMP1 in mediating energy metabolism. Notably, LMP1-induced upregulation of HK2 conferred NPC cells with a proliferative advantage and a high level of HK2 expression was shown to correlate with LMP1 expression and poor overall survival of NPC patients following radiation therapy. Notably, knockdown of HK2 expression overcame the resistance of LMP1-expressing NPC cells to radiation treatment. In addition, we found c-Myc to be a key transcriptional factor of LMP1-mediated upregulation of HK2; and LMP1-mediated attenuation of the PI3-K/Akt-GSK3beta-FBW7 signaling axis results in stabilization of c-Myc.

With the rapid progress in the area of large-scale profiling analysis (for example, genomics, proteomics and metabolomics), deciphering the full scope of pathogen-induced deregulation of metabolism is now possible. These technologies can provide fundamental insights into the basic biochemistry of the oncogenic virus-induced tumorigenic effects and lead to the discovery of potential opportunities in clinical treatment for infection-related cancers. For cancer cells to sustain their rapid proliferation and gain a survival advantage, glycolysis has been demonstrated as a hot spot for infection-mediated metabolic reprogramming.^19, 20, 21, 22, 23^ We found that LMP1 could mediate the upregulation of several glycolytic genes, suggesting that oncogenic viruses might have evolved similar mechanisms to hijack critical cellular interaction networks and lead to the increased amounts of glucose delivery to proliferating cells to enhance their tumorigenic capacity. In turn, the upregulated glycolysis is also able to influence the viral infection. For instance, high glucose concentrations were reported to induce a dose-specific increase in influenza infection, and viral replication was significantly reduced after cells were treated with glycolytic inhibitors.^24^ Furthermore, the elevated glycolytic level in Kaposi's sarcoma-associated herpesvirus infection was demonstrated to be necessary for maintaining Kaposi's sarcoma-associated herpesvirus latently infected cells.^21^ Similar reports are numerous and overall indicate the important role of metabolic modification in viral infection. Given the importance of oncogenic viruses and their encoded oncogenes in the deregulation of glucose metabolism, manipulating glycolytic genes or gene products for improving treatment of infection- related cancers seems plausible.

By intensively investigating the biological function of metabolic genes in cancer cells, scientists have discovered some of these genes, such as *pyruvate kinase M2* and *isocitrate dehydrogenases*, to be able to perform some unexpected but critical functions that differ from their primary role in metabolism.^25,26^ Herein we found that HK2 could not only potently induce glycolysis, but also significantly inhibit mitochondrial-dependent apoptosis, which is vital for LMP1-mediated radiation resistance. HK2 has been demonstrated to be required for tumor initiation and maintenance, and it has been recognized as a therapeutic target in multiple cancers.^27^ Recently, two NPC clinical investigations reported that high ^18^F-FDG uptake indicates poor outcome in patients with NPC,^28,29^ and the high ^18^F-FDG uptake is highly dependent on HK2. Collectively, we suggest that therapeutic strategies targeting multifunctional metabolic genes, such as *hk2*, would provide more effective treatment options for NPC.

Even though the underlying mechanisms of the deregulation of glucose metabolism in cancer cells have not yet been completely elucidated, a number of tumor-related genes, such as *c-myc* and *hypoxia inducible factors-1α (HIF-1α)*, are known to participate in modulating energy metabolic reprogramming in cancer cells.^30^ Most factors that are crucial for maintaining the cancer metabolic phenotype have been reported to consistently alter the PI3-K/Akt-related signaling pathway. For example, in PTEN transgenic mouse, PTEN overexpression results in decreased c-Myc levels, mimics an ‘anti-Warburg effect state' and confers cancer resistance *in vivo* by negatively regulating the PI3-K/Akt-dependent pathway.^31^ This highlights the relationship between PI3-K/Akt and c-Myc in metabolic reprogramming in cancer.^32^

FBW7 is an E3 ligase with tumor suppressor function in multiple cancers, and protein products of oncogenes, which have been phosphorylated by GSK3beta, can be recognized by FBW7, and then degraded in a ubquitination-dependent manner.^15^ Reportedly, FBW7 is rarely mutated in head and neck cancer, and its tumor suppressor function in these cancer cells is more dependent on the activity of GSK3beta.^33^ Previous studies reported that LMP1 increased the protein level of c-Myc, activated the PI3-K/Akt signaling pathway and inhibited the activity of GSK3beta through the large cytosolic C terminus of LMP1, eventually facilitating the growth and migration of host cells.^34,35^ Recently, the localization of LMP1 to lipid rafts has been reported to induce the localization and activation of PI3-K/Akt.^36^ Regarding the mechanism of LMP1-induced c-Myc upregulation, we found that LMP1-mediated activation of PI3-K/Akt decreased the activity of the GSK3beta-FBW7 signaling axis and stabilized c-Myc at the protein level. Moreover, either knocking down c-Myc expression or inhibiting PI3-K/Akt was able to reduce LMP1-mediated upregulation of HK2, and inhibit LMP1-augmented glycolysis. Besides c-Myc, HIF-1a is another important transcriptional factor responsible for the transcription of HK2,^37^ and its stability can also be regulated by LMP1 in EBV-related cancers.^38,39^ LMP1-mediated upregulation of HIF-1a might also contribute to the transactivation of HK2. In addition, we found that LMP1 increased the interaction between VDAC and HK2. Previous studies demonstrated that to promote apoptosis, activated GSK3 could inhibit the interaction between VDAC and HK2.^40,41^ These findings uncover a previously unknown mechanism of c-Myc upregulation by LMP1 and identify a PI3-K/Akt-GSK3beta-FBW7-c-Myc signaling axis that enhances glycolysis in the presence of LMP1 in NPC cells.

**Figure 8:**
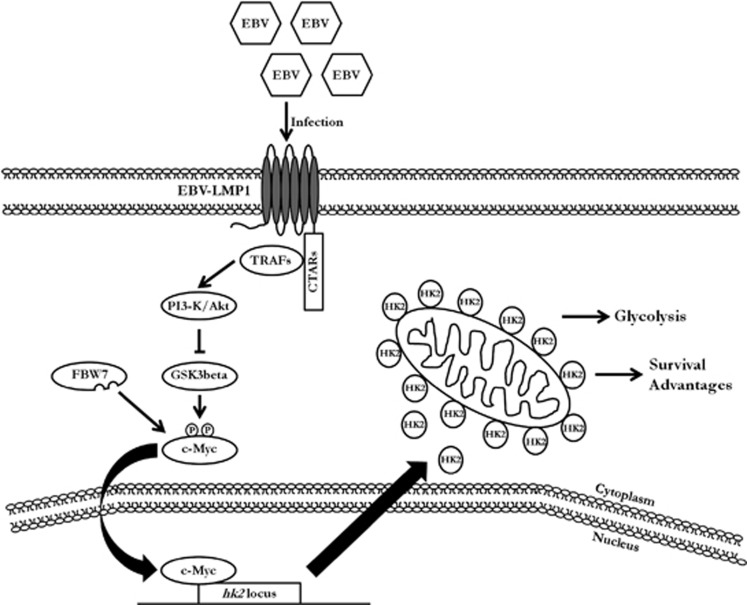
A schematic for reprogramming of EBV-induced glucose metabolism. The PI3-K/Akt signaling pathway likely has critical roles in EBV-mediated glucose metabolism reprogramming, which acts mainly by disrupting the stabilization of c-Myc. When the PI3-K/Akt-GSK3beta-FBW7-c-Myc signaling axis is impeded, c-Myc transactivates the transcription of HK2, causing the upregulation of glycolysis and confers resistance against mitochondrial-dependent apoptosis, which correlates with the poor overall survival of NPC patients.

In summary, this study illustrates the interaction between metabolic changes and EBV infection. The ability of LMP1 to induce glycolytic enzymes contributes to the significance of EBV in cellular energy metabolism, especially in glucose metabolism in NPC cells. HK2 was suggested to promote malignant proliferation, inhibit apoptosis and especially cause resistance of NPC cells to radiation therapy, leading to the poor overall survival of NPC patients. We propose a scheme (Figure 8) for EBV-induced metabolic reprogramming and proliferation and resistance of NPC cells to apoptosis. HK2 is considered as a vital modulator of this phenotype. With these discoveries, we could more fully understand infection-mediated carcinogenesis and provide a novel target to improve the therapeutic regimen against NPC.

## Materials and methods

### Cell lines and culture conditions

The immortalized NP69 nasopharyngeal epithelial cell line was cultured in keratinocyte serum-free medium (Invitrogen, Carlsbad, CA, USA). The NPC cell lines, including C666-1, C666-1-shLMP1, CNE1, CNE1-LMP1, HNE2, HNE2-LMP1, HK1, HK1-LMP1, SUNE1, SUNE1-LMP1, HNE1, were cultured in RPMI-1640 medium (Hyclone, Logan, UT, USA) supplemented with 10% fetal bovine serum (Hyclone). The EBV-positive Burkitt's lymphoma cell line P3HR1 (ATCC HTB-26) was cultured in RPMI-1640 medium (Hyclone) supplemented with 20% fetal bovine serum (Hyclone). 293 [HEK293] (ATCC CRL1573), LMP1 tet-on HEK293 and 293T/17 (HEK 293T/17) cells were maintained in Dulbecco's Modified Eagle Medium (Hyclone) supplemented with 10% fetal bovine serum.

### Metabolic profiling

Metabolomic profiles were obtained to assess the relative distribution of various cellular metabolites of immortalized nasopharyngeal epithelial cell and NPC cells. Cells were collected and quickly frozen. Further sample preparation, metabolic profiling, peak identification and curation were performed by Metabolon (Durham, NC, USA) using their described methods.^42^

### Measurement of glucose uptake and lactate production

Cells (5 × 10^5^) were seeded in 6-well plates and after incubation for 6 h, media were discarded and then cells were incubated in fresh media for another 8 h. Glucose and lactate levels were measured using the Automatic Biochemical Analyzer (AU680, Beckman Coulter International, Brea, CA, USA) at the Clinical Biochemical Laboratory of Xiangya Hospital (Changsha, China). The relative glucose consumption rate and lactate production rate were normalized by the protein concentration of samples.

### qRT–PCR screening

Cellular RNA was isolated and converted into cDNA. qRT–PCR was performed using ABI-7500 (Applied Biosystems, Fostercity, CA, USA) by mixing equal amounts of cDNAs, iQ SYBR Green Supermix (Bio-Rad, Hercules, CA, USA) and specific primers. All real-time data were normalized to β-actin. The primers used in qRT–PCR screening are shown in Supplementary Table 2.

### Western blot analysis and antibodies

After electrophoretic separation and immunoblotting of whole-cell lysates, blots were incubated with appropriate primary antibodies and secondary antibodies labeled with horseradish peroxidase. Visualization of proteins was performed using the ChemiDoc XRS system with Image Lab software (Bio-Rad). Antibodies to detect HK2, phosphorylated Akt (Ser473), PARP, caspase-9, c-Myc, phosphorylated GSK3beta (Ser9) were obtained from Cell Signaling Technologies (Danvers, MA, USA). The antibodies against β-actin and LMP1 were from Sigma-Aldrich (St Louis, MO, USA) and DAKO (Glostrup, Denmark), respectively. Anti-α-tubulin, anti-mouse immunoglobulin, anti-rabbit immunoglobulin and normal rabbit immunoglobulin were purchased from Santa Cruz Biotechnology (Santa Cruz, CA, USA). Anti-VDAC, phosphorylated Myc (Thr58) and FBW7 were purchased from Abcam (Cambridge, UK).

### Plasmids

The psg5-based expression vector for LMP1 that was derived from the B95.8 EBV strain was kindly provided by Dr Kenneth M Izumi (Brigham and Women's Hospital, Boston, MA, USA). Target sequences from the RNA interference Consortium shRNA Library were used to construct scrambled shRNA and shRNA-targeting HK2. The target sequence for constructing shRNA targeting c-Myc came from Haining Yang *et al.*^43^ and the target sequence for constructing shRNA targeting LMP1 was from Aron R Marquitz *et al.*^44^ The specific sequences (as shown in Supplementary Table 3) were cloned into the pLKO.1 vector or pLenti6/Block-iT-DEST vector.

### Cell viability assay

Cells were cultured in 96-well plates. Cell viability was examined using the CellTiter 96 Aqueous One Solution (MTS) Reagent (Promega, Fitchburg, WI, USA) according to the manufacturer's instructions.

### Immnunohistochemistry analysis of an NPC tissue array and NPC biopsies

The NPC tissue array was purchased from Pantomics (Richmond, CA, USA) and follow-up data and clinical details were provided by this company and are available online. NPC biopsies, verified by pathologist Dr Bo Li (Xiangya Hospital), were obtained from the Pathology Department of Xiangya Hospital. Immunohistochemistry was performed as previously described.^45^ Images of the sections were acquired and differentially quantified by two pathologists, Dr Bo Li and Dr Songqing Fan (The Second Xiangya Hospital, Changsha, China).

### Colony formation and radiosensitivity assays

NPC cells were seeded at 100 cells/well in six-well plates in triplicate. After 10–12 days, cell clones were washed three times with phosphate-buffered saline, fixed in methanol for 10 min and then stained with crystal violet for 10 min at room temperature. Colonies containing more than 50 cells were counted and the surviving fractions were calculated. The radiosensitivity assay was described previously.^8^ Briefly, cells were counted and seeded in six-well plates at densities of 1 × 10^2^, 4 × 10^2^, 1 × 10^3^ or 1 × 10^4^ per well. Cells were treated once with radiation (0, 2, 4 or 6 Gy) and then the colony formation assay was conducted. Colonies containing more than 50 cells were counted and the surviving fractions calculated. The linear quadratic model was utilized for mathematic analysis of radiosensitivity of cells using GraphPad Prism (GraphPad Software, La Jolla, CA, USA).

### Chromatin Immunoprecipitation

ChIP was performed using the ChIP assay kit (Upstate Biotechnology, Lake Placid, NY, USA) according to the manufacturer's recommendations. The antibodies used were c-Myc and rabbit immunoglobulin. The primers from the *hk2* promoter domain containing the c-Myc-binding region used in the ChIP assays are shown in Supplementary Table 1.

### Protein stability analysis

For protein stability experiments, 20 μg/ml cycloheximide were added to cells to inhibit protein synthesis. Cells were incubated in medium containing cycloheximide for 0, 20, 40, 60, 80 or 100 min as indicated and then cells were processed as described above for western blotting.

### Statistical analysis

The Kaplan–Meier method was used to estimate overall survival and the log-rank test was used to evaluate differences between survival curves. Statistical analyses were performed using the Student *t*-test or Welch's *t*-test (normal distribution). A *P*-value of <0.05 was considered statistically significant.
