# Supplementary figures and images for: Targeting Epstein–Barr virus oncoprotein LMP1-mediated glycolysis sensitizes nasopharyngeal carcinoma to radiation therapy
Source: Oncogene. 2014 Mar 24;33(37):4568–78. doi: 10.1038/onc.2014.32 (PMC4162460; doi:10.1038/onc.2014.32)

# Supplemental Figure 1

**A**

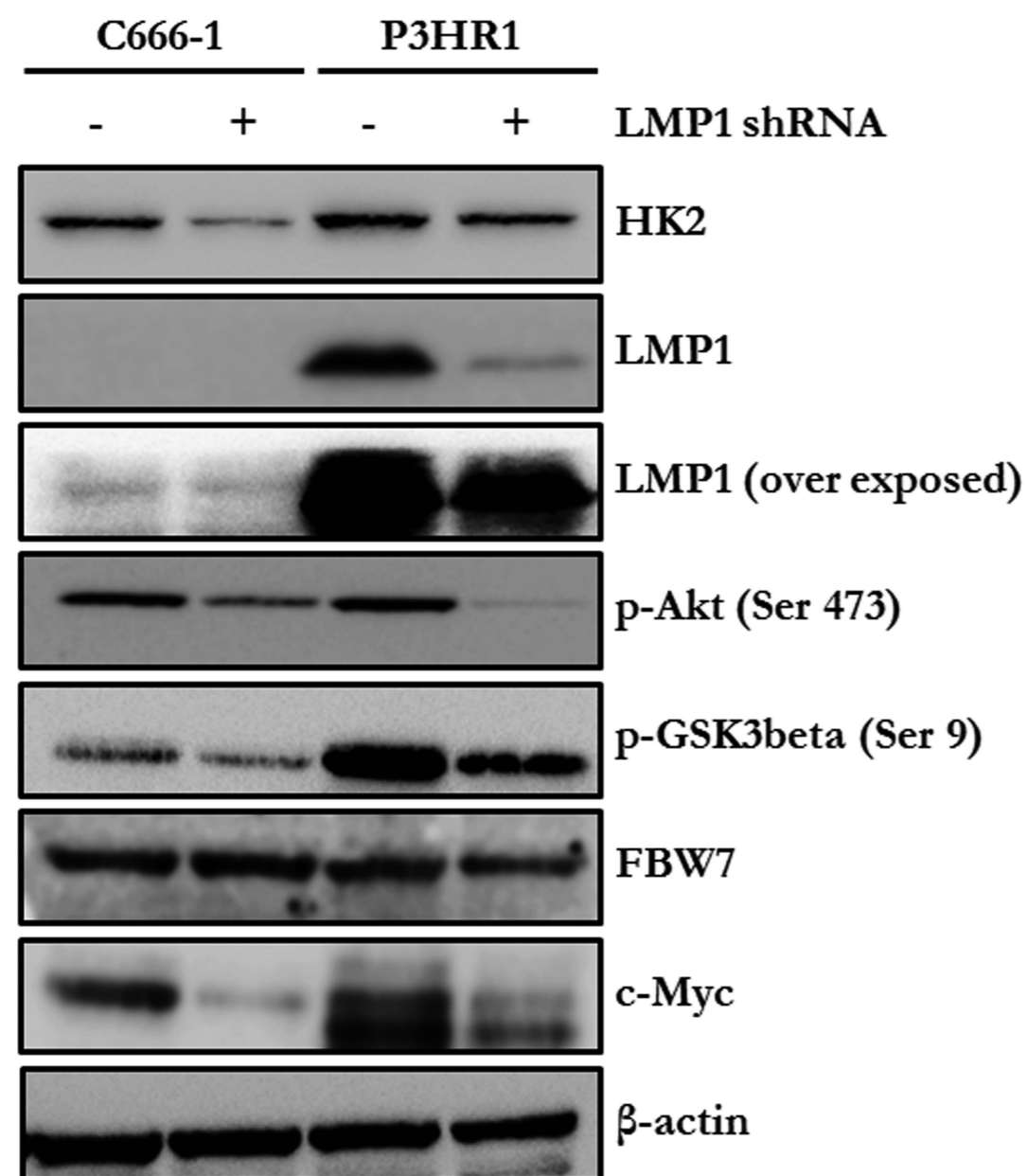

**B**

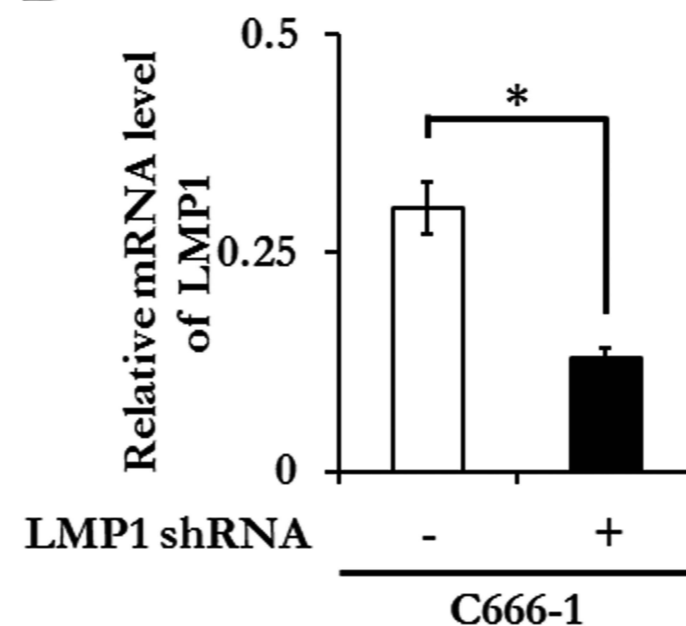

**C**

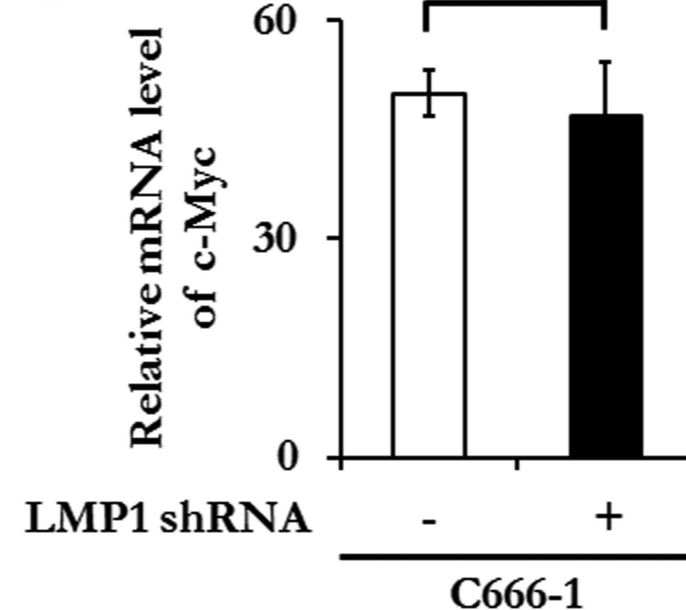

**D**

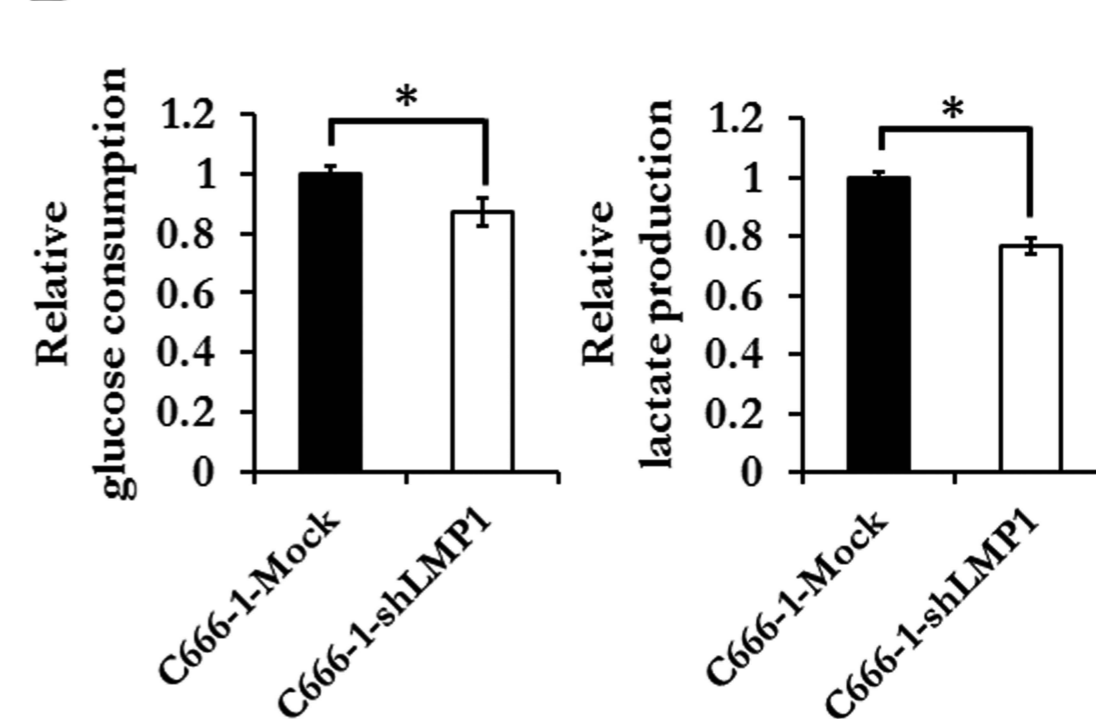

**E**

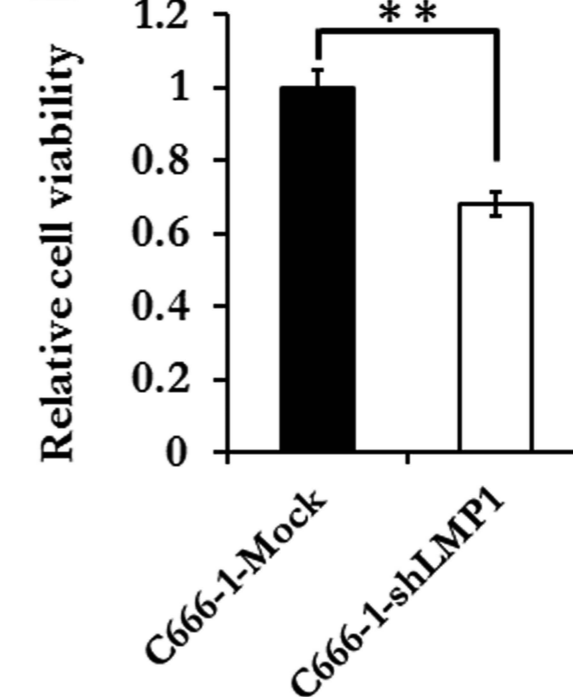

Supplement: Supplementary Figure S1 [file onc201432x1.pdf]

# Supplemental Figure 2

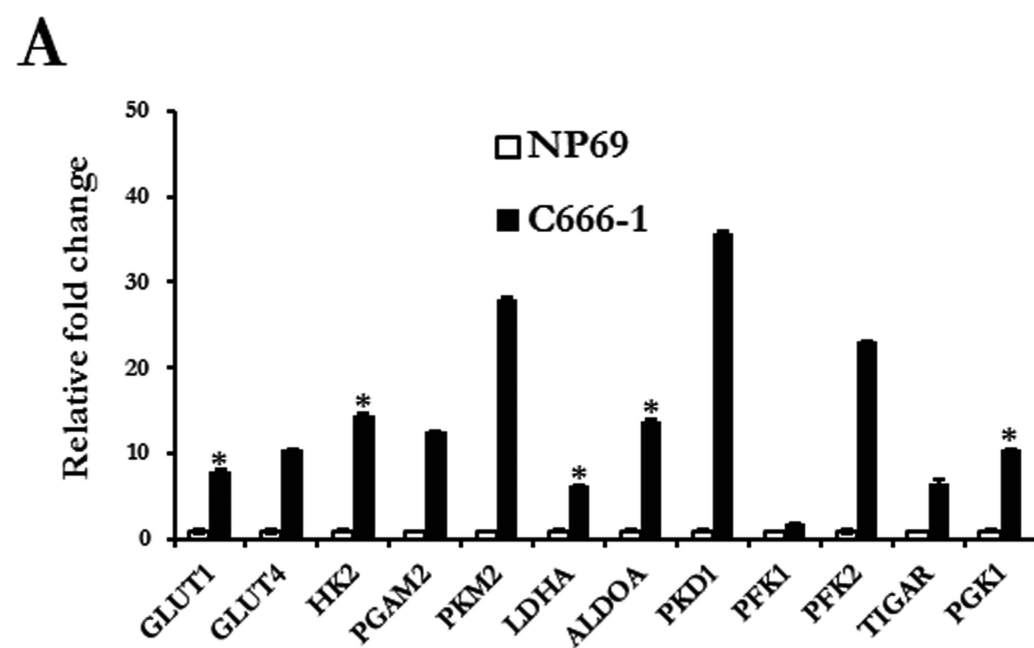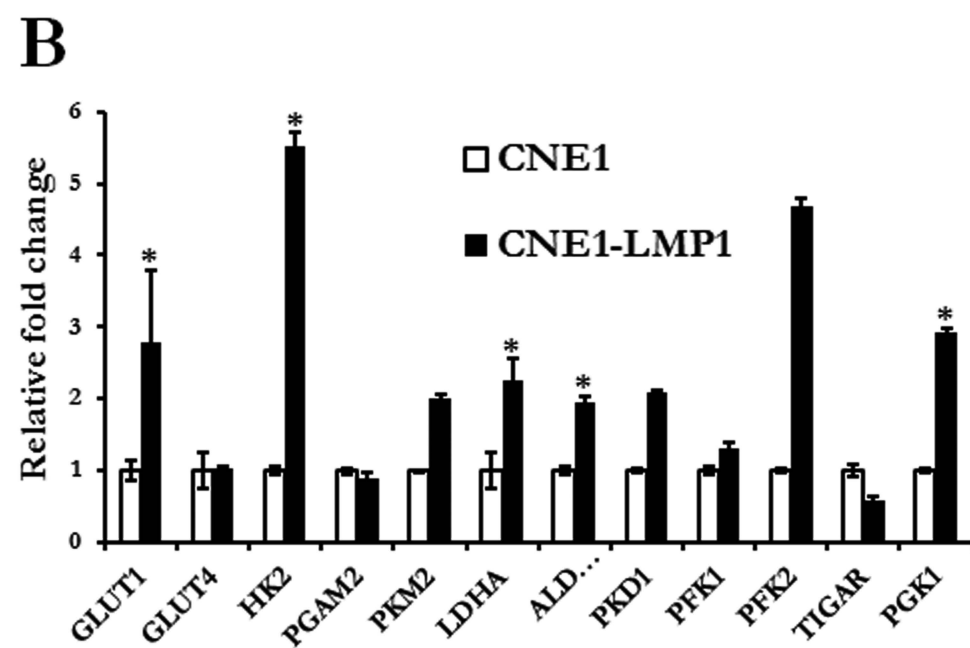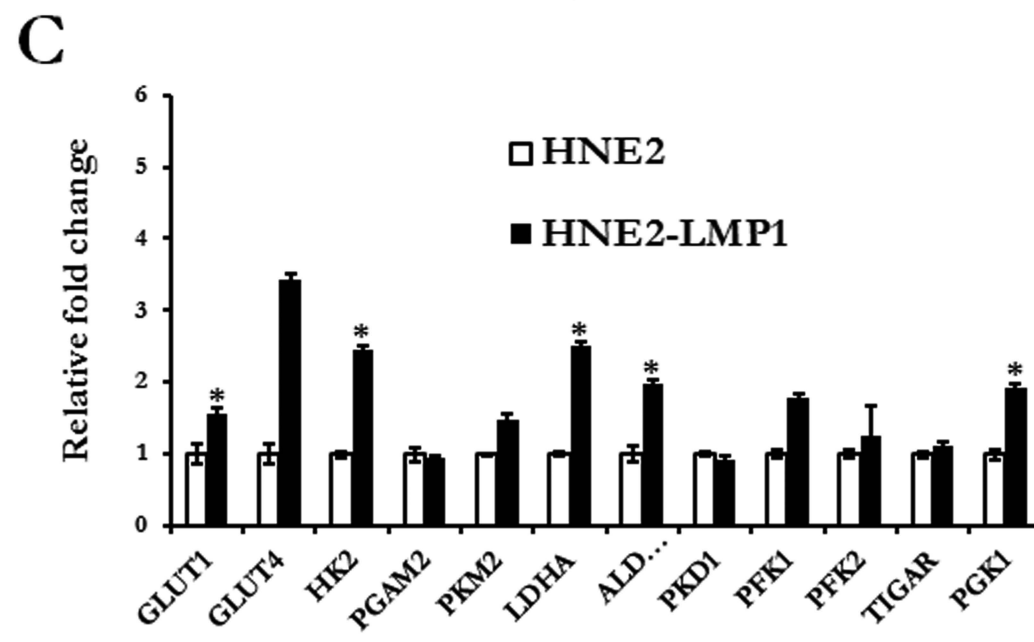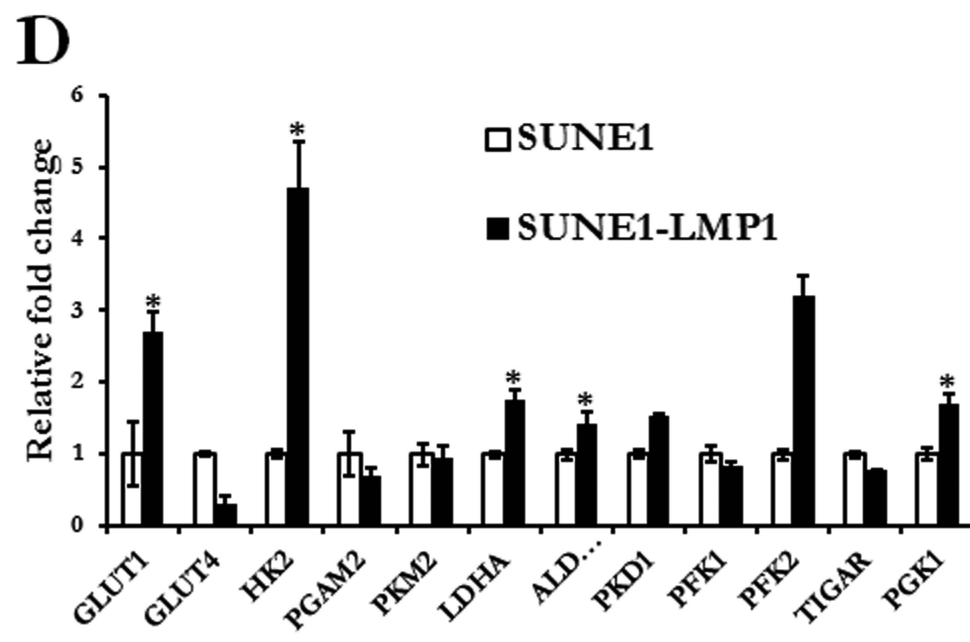

Supplement: Supplementary Figure S2 [file onc201432x2.pdf]

Supplemental Figure 4

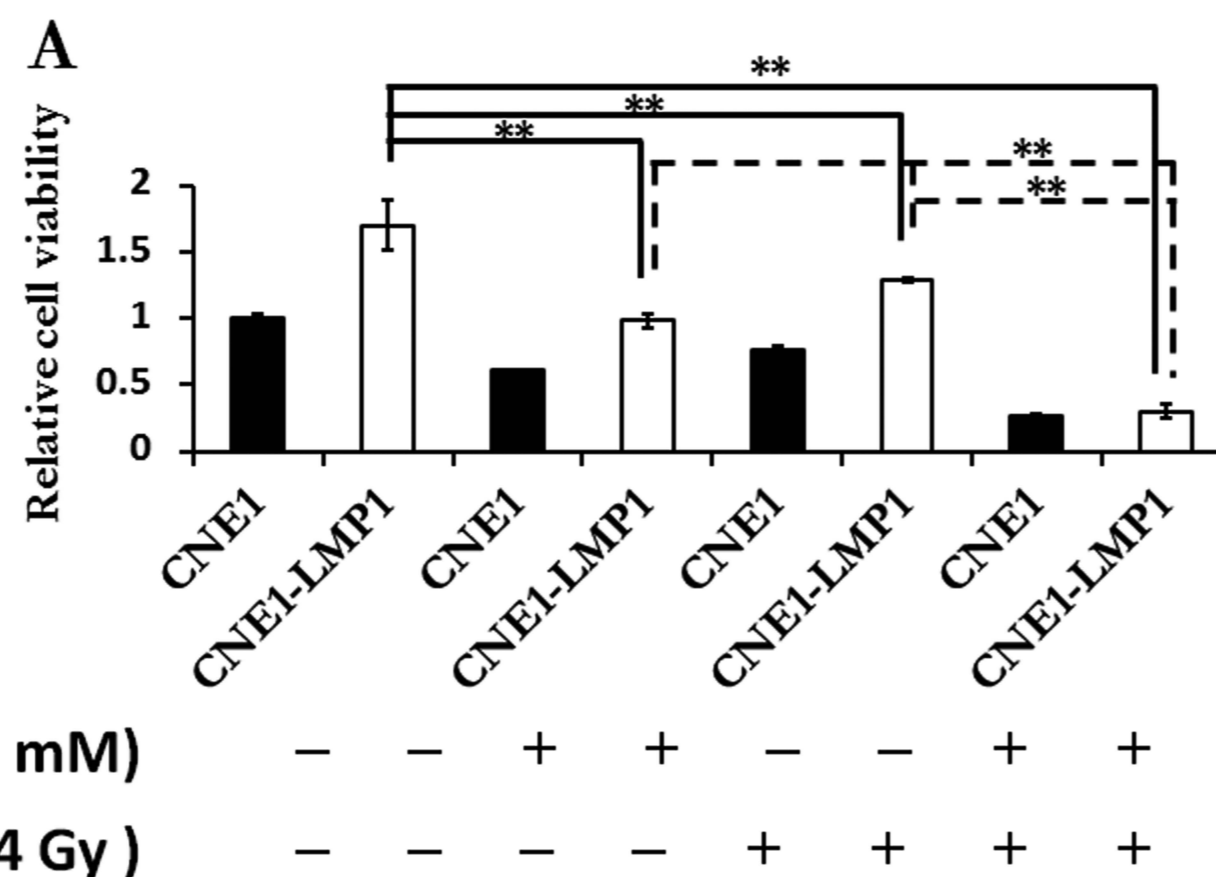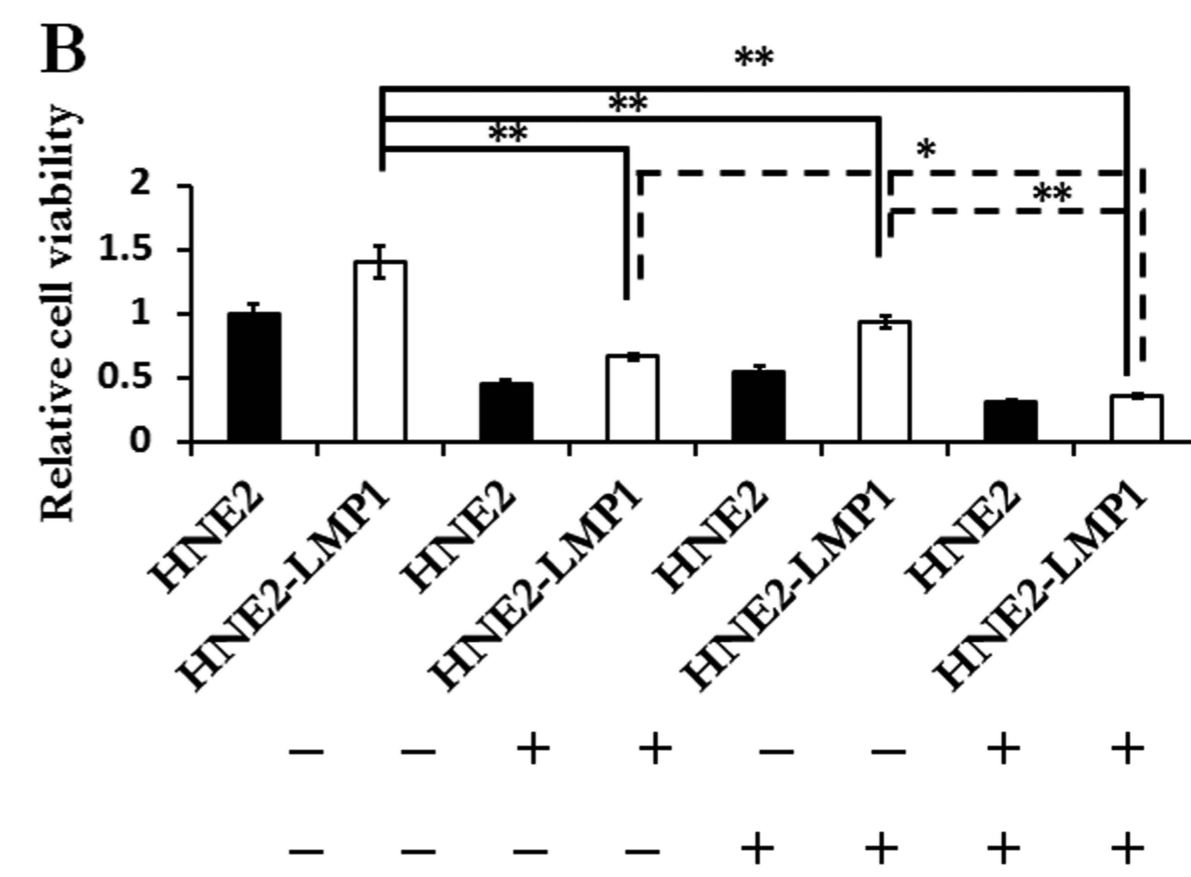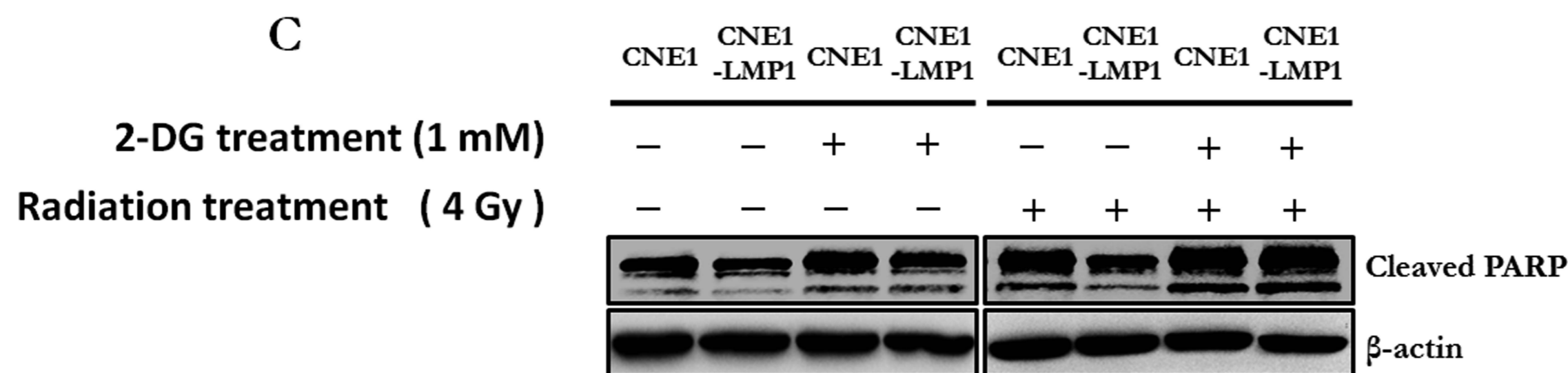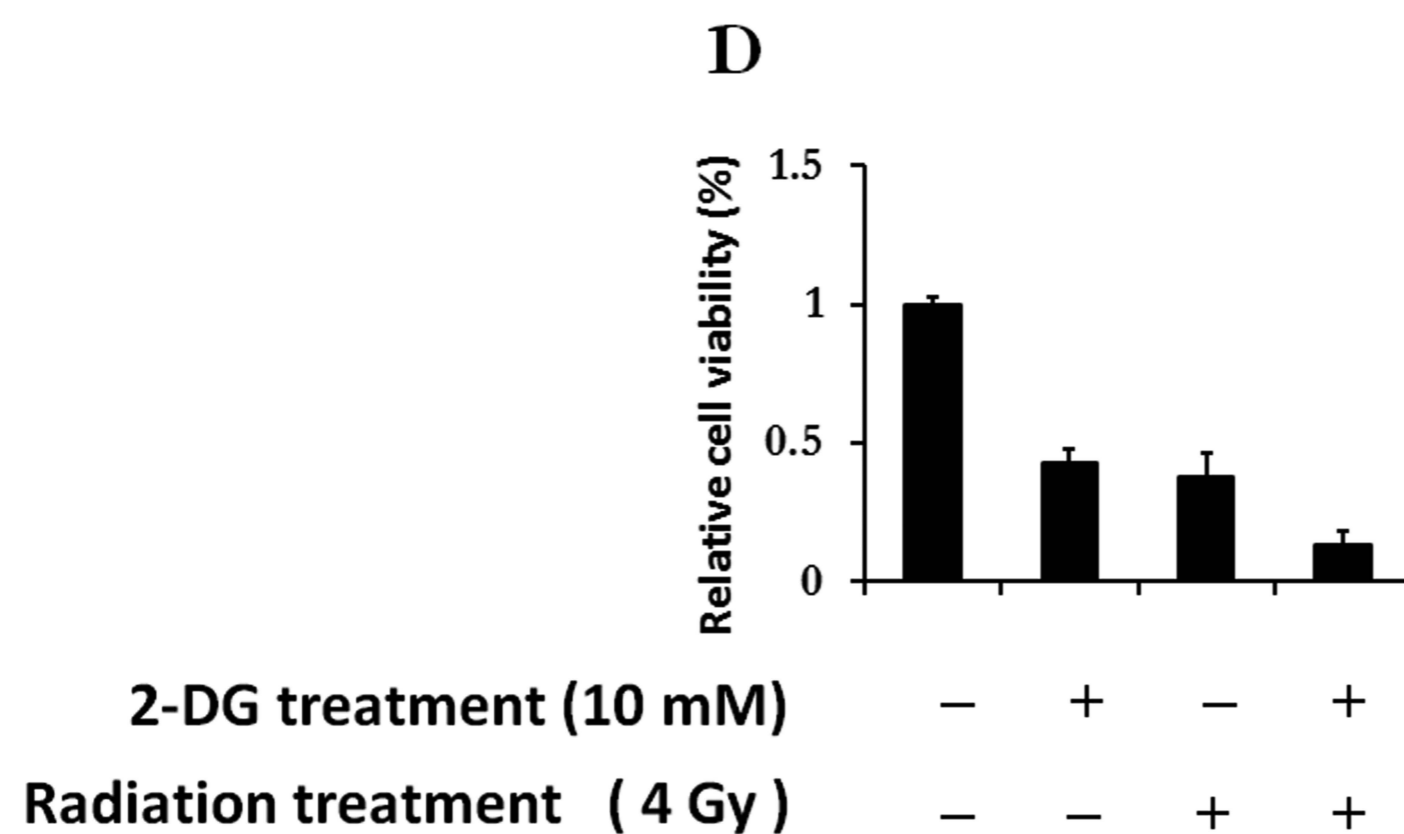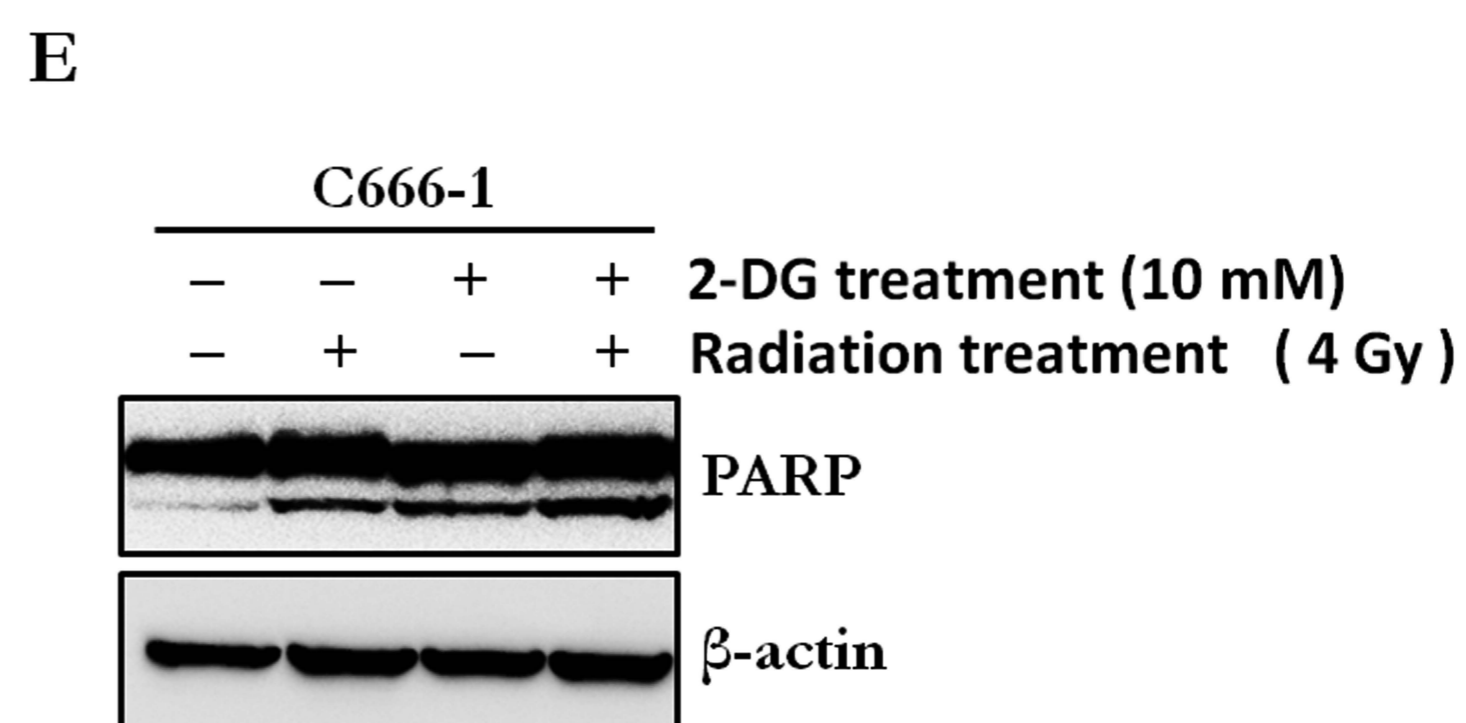

Supplement: Supplementary Figure S4 [file onc201432x4.pdf]

# Supplemental Figure 5

A

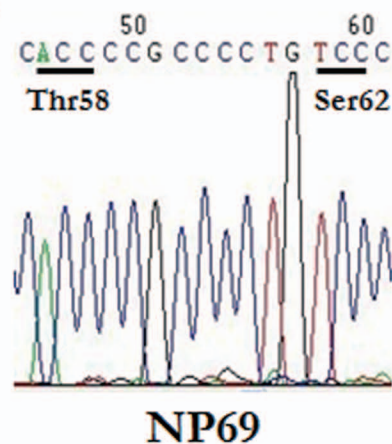

B

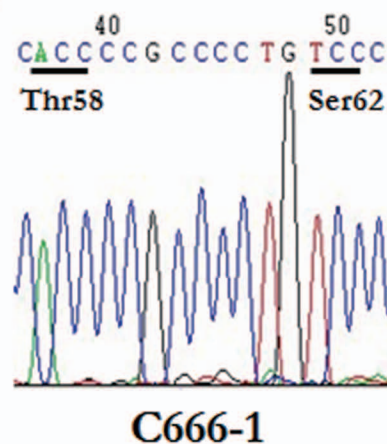

C

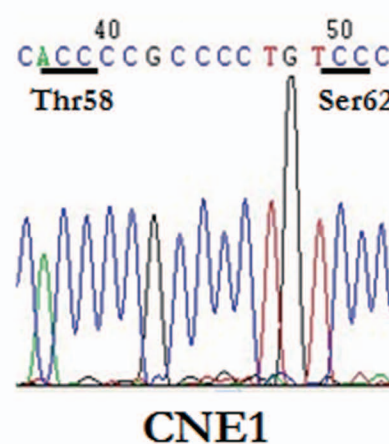

D

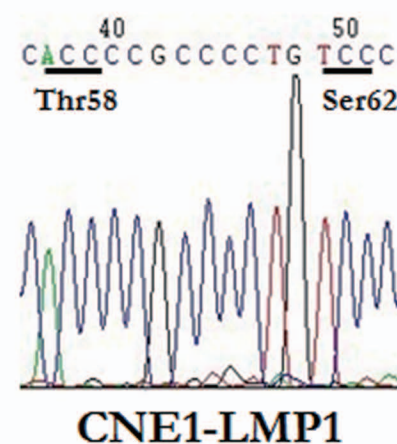

E

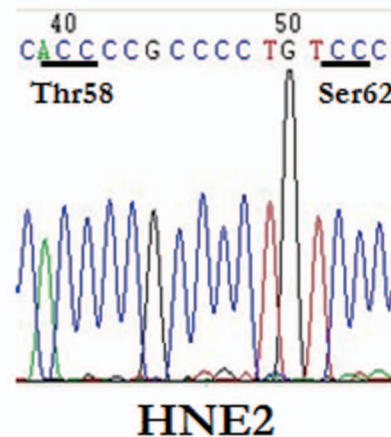

F

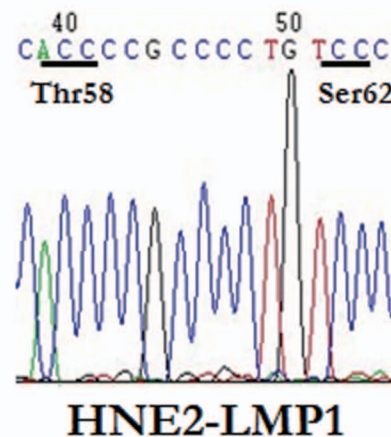

Supplement: Supplementary Figure S5 [file onc201432x5.pdf]
