# Supplementary material for: Targeting Epstein–Barr virus oncoprotein LMP1-mediated glycolysis sensitizes nasopharyngeal carcinoma to radiation therapy
Source: Oncogene. 2014 Mar 24;33(37):4568–78. doi: 10.1038/onc.2014.32 (PMC4162460; doi:10.1038/onc.2014.32)

Supplemental Figure 3

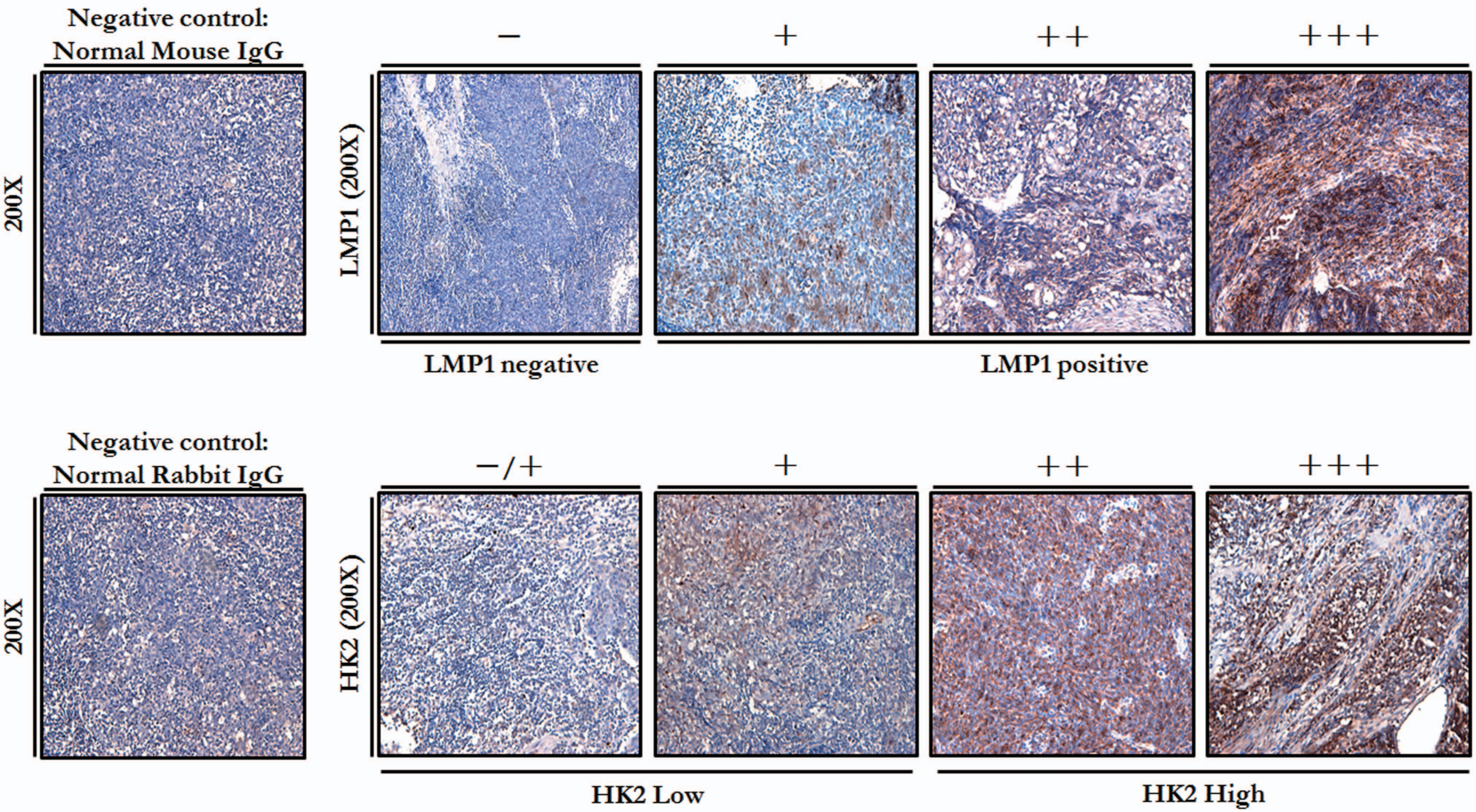

Supplement: Supplementary Figure S3 [file onc201432x3.pdf]
