# Supplementary material for: Targeting Epstein–Barr virus oncoprotein LMP1-mediated glycolysis sensitizes nasopharyngeal carcinoma to radiation therapy
Source: Oncogene. 2014 Mar 24;33(37):4568–78. doi: 10.1038/onc.2014.32 (PMC4162460; doi:10.1038/onc.2014.32)
Supplement: Supplementary Information [file onc201432x6.doc]

**Supplemental materials.**

Summary: In supplemental materials, we provide the primers used in this paper, the sequences used to construct shRNAs, qRT-PCR screening of glycolytic genes, results of knockdown of LMP1 in C666-1 and P3HR1 cell, characteristics of NPC tissue array, the relative IHC quantitative standard of HK2 and LMP1, the 2-DG IC50 of NPC cells, the effects of irradiation alone, 2-DG treatment alone or irradiation combined with 2-DG treatment in NPC cells and the mutation statues of c-Myc at Thr58 and Ser62 of all tested cells.

**1. Supplemental Figure 2. Knockdown of LMP1 in C666-1 and P3HR1 cell.**

A. Immunoblot analysis was performed to analyze the expression level of HK2, LMP1, p-Akt, p-GSK3Beta, FBW7 and c-Myc in C666-1-shLMP1 and P3HR1-shLMP1 cells compared with their parental counterparts. β-Actin was used as a control to confirm equal loading of protein.

B and C. qRT-PCR analysis was performed to detect the mRNA of LMP1 and c-Myc in C666-1-mock and C666-1-shLMP1 cells. β-Actin was used as a control to confirm equal loading of cDNAs. Data are shown as mean ± S.D. of 3 experiments. * *p* < 0.05 and NS means none of statistical significant.

D. The relative glucose consumption rate and lactate production rate were examined in C666-1-mock and C666-1-shLMP1 cells. Data are shown as mean ± S.D. of 3 experiments. * *p* < 0.05. E. MTS assay was performed to determine the relative cell viability of C666-1-Mock and C666-1-shLMP1 cells. Data are shown as mean ± S.D. of 3 experiments. ** *p* < 0.001.

**2**. Supplemental Table 1. The primers used for qRT-PCR screening, qChIP and Myc sequencing

| **Gene** | **Forward** | **Reverse** |
| --- | --- | --- |
| ***glut1*** | 5’-GTCACCATCCTGGAGCTGTT-3’ | 5’-GAAGGCCGTGTTGACGATAC-3’ |
| ***glut4*** | 5’-TGGGCGGCATGATTTCCTC-3’ | 5’-GCCAGGACATTGTTGACCAG-3’ |
| ***hk2*** | 5’-GAGCCACCACTCACCCTACT-3’ | 5’-CCAGGCATTCGGCAATGTG-3’ |
| ***pgam2*** | 5’-AGAAGCACCCCTACTACAACTC-3’ | 5’-TCTGGGGAACAATCTCCTCGT-3’ |
| ***pkm2*** | 5’-ATGTCGAAGCCCCATAGTGAA-3’ | 5’-TGGGTGGTGAATCAATGTCCA-3’ |
| ***ldha*** | 5’-TTGACCTACGTGGCTTGGAAG-3’ | 5’-GGTAACGGAATCGGGCTGAAT-3’ |
| ***alda*** | 5’-ATGCCCTACCAATATCCAGCA-3’ | 5’-GCTCCCAGTGGACTCATCTG-3’ |
| ***pkd1*** | 5’-GGATCGGCTTCTCGACTGTG-3’ | 5’-ACGAGGAGGTTCTCGGCAT-3’ |
| ***pfk1*** | 5’-GGTGCCCGTGTCTTCTTTGT-3’ | 5’-AAGCATCATCGAAACGCTCTC-3’ |
| ***pdfk2*** | 5’-TTGGCGTCCCCACAAAAGT-3’ | 5’-AGTTGTAGGAGCTGTACTGCTT-3’ |
| ***tigar*** | 5’-TAGTTGTGAGTCACGGTGCTT-3’ | 5’-GCGAGTTTCAGTCAGTCCATT-3’ |
| ***lmp1*** | 5’-CGTTATGAGTGACTGGACTGGA-3’ | 5’-TGAACAGCACAATTCCAAGG-3’ |
| ***β-actin*** | 5’-CATGTACGTTGCTATCCAGGC-3’ | 5’-CTCCTTAATGTCACGCACGAT-3’ |
| ***Chip-Myc-hk2*** | 5’-CCCAGTCCCTTTTTCCCTGTT-3’ | 5’-GAAGGAGAAGGGAACCGCTCG-3’ |
| ***Seq-Myc*** | 5’-GAACTTCTACCAGCAGCAGCA-3’ | 5’-AGAAGCCGCTCCACATACAGT-3’ |

**Note:** Primers shown in Supplemental Table 1 were used to investigate changes in glucose metabolism-related genes involved in the LMP1-mediated disruption of glucose metabolic reprogramming, to examine c-Myc binding to the HK2 locus, and sequence the mutation state of c-Myc in cells.

**3. Supplemental Table 2. The sequences used for HK2, c-Myc and LMP1 shRNA constructions.**

| **shRNA** | **Sequence** |
| --- | --- |
| **pLKO.1-shHK2#1** | **5’-CCGGCCATCTCCTGTCCAATGACATCTCGAGATGTCATTGGACAGGAGATGGTTTTT-3’** |
| **pLKO.1-shHK2#2** | **5’-CCGGCACAACCTGTTTGAGCCTGAACTCGAGTTCAGGCTCAAACAGGTTGTGTTTTT-3’** |
| **pLKO.1-shHK2#3** | **5’-CCGGTGCTAGAGCTTACTCTGAGAACTCGAGTTCTCAGAGTAAGCTCTAGCATTTTT-3’** |
| **pLKO.1-shc-Myc** | **5’-CCGGTGACGAGAACAGTTGAAACACTCGAGTGTTTCAACTGTTCTCGTCTTTTT-3’** |
| **pLenti6/Block-LMP1** | **5’-GAATTTGCACGGACAGGCCGAAGCCTGTCCGTGCAAATTCC-3’** |

**4. Supplemental Figure 2. The EBV and LMP1-mediated deregulation of glycolytic genes.** To investigate the molecular mechanism of LMP1-mediated elevation of glycolysis, qRT-PCR screening several glytolytic genes was performed in NP69 and NPC cell lines (CNE1 and CNE1-LMP1, and HNE2 and HNE2-LMP1 cells). β-Actin was used as a control to confirm equal loading of cDNAs. Data are shown as mean ± S.D. of 3 experiments. * *p* < 0.05 and ** *p* < 0.001.

**5. Supplemental Table 3. Clinical characteristics of NPC patients from an NPC tissue array**

| **Characteristic** | **Number of patients (%)** |
| --- | --- |
| **Sex** |  |
| **Male** | **38 (80.85%)** |
| **Female** | **9 (19.15%)** |
| **Age (years)** |  |
| **Median (range)** | **43** |
| **> 43** | **21 (48.90%)** |
| **≤ 43** | **27 (51.10%)** |
| **Follow-up time (years)** |  |
| **Average** | **5.81** |
| **Clinical Stage** |  |
| **Ⅰ** | **1 (2.13%)** |
| **Ⅱ~Ⅲ** | **18 (38.30%)** |
| **Ⅲ** | **28 (59.57%)** |
| **Therapeutic modality** |  |
| **Radiation Therapy** | **36 (76.59%)** |
| **Concomitant chemoradiaotherapy** | **5 (10.64%)** |
| **None treatment** | **6 (12.77%)** |
| **Who histological classification** |  |
| **NKUC** | **45 (95.74%)** |
| **KSCC** | **2 (4.26%)** |
| **OS rate (%) 5 year** | **53.19%** |

**Note:** In the NPC tissue array, 47 patients were successfully followed up. The patients' clinical characteristics are listed.

**6. Supplemental Figure 3. The relative IHC quantitative standard of HK2 and LMP1.** Normal mouse IgG and normal rabbit IgG severed as negative control of LMP1 and HK2 respectively. The relative quantitation of HK2 and LMP1 are according to the relative quantitative standard of HK2 and LMP1 respectively.

**7. Supplemental Table 4. The 2-DG IC50 of NPC cells.**

| **Cell Line** | **IC50 (mM)** | ***p* value** |
| --- | --- | --- |
| **C666-1-Mock** | **4.237 ( ±0.607)** | **1.27E-02** |
| **C666-1-shLMP1** | **9.922 ( ±2.267)** |
| **CNE1** | **1.399 ( ±0.386)** | **9.88E-03** |
| **CNE1-LMP1** | **0.286 ( ±0.162)** |
| **HNE2** | **1.337 ( ±0.390)** | **2.06E-02** |
| **HNE2-LMP1** | **0.372 ( ±0.204)** |

**Note:** Statistical analyses were performed using the Student t-test. A *p* value of < 0.05 was considered statistically significant.

**8. Supplemental Figure 4.** The Effects of irradiation alone, 2-DG treatment alone or irradiation combined with 2-DG treatment in NPC cells.

A and B. Effects of irradiation alone, 2-DG treatment alone or irradiation combined with 2-DG treatment were measured by MTS assay. For combination treatment, CNE1, CNE1-LMP1, HNE2 and HNE2-LMP1 cells were treated with 2-DG (1 mM) and then treated with radiation. The relative inhibition was calculated by comparing the OD value of each treatment group with control group. Data are shown as mean ± S.D. of 3 experiments. * *p* < 0.05 and ** *p* < 0.001.

C. Immunoblotting analysis was performed to detect the level PARP (totoal and cleaved PARP) in CNE1 and CNE1-LMP1 cells treating 2-DG or not and exposed or not to radiation treatment. β-Actin served as internal control to confirm equal protein loading.

D. Effect of radiation treatment combined with 2-DG treatment was measured by MTS assay. C666-1 cells were treated with 2-DG (10 mM) and then treated with radiation. The relative inhibition was calculated by comparing the OD value of each treatment group with control group. Data are shown as mean ± S.D. of 3 experiments. * *p* < 0.05 and ** *p* < 0.001.

E. Immunoblotting analysis was performed to detect the level PARP (totoal and cleaved PARP) in C666-1 cells treating 2-DG or not and exposed or not to radiation treatment. β-Actin served as internal control to confirm equal protein loading.

**9. Supplemental Figure 5.** The mutation statues of c-Myc in immortalization nasopharyngeal epithelial cells and NPC cells. Genomic DNA of immortalization nasopharyngeal epithelial cells and NPC cells were used to PCR and then sequenced the Thr58 and Ser62 of c-Myc in all tested cells.
